# Proteomic Signatures of Thymomas

**DOI:** 10.1371/journal.pone.0166494

**Published:** 2016-11-10

**Authors:** Linan Wang, Owen E. Branson, Konstantin Shilo, Charles L. Hitchcock, Michael A. Freitas

**Affiliations:** 1 Department of Cancer Biology and Genetics, The Ohio State University Wexner Medical Center, Columbus, OH, United States of America; 2 The Ohio State University Comprehensive Cancer Center, Columbus, OH, United States of America; 3 Department of Pathology, The Ohio State University Wexner Medical Center, Columbus, OH, United States of America; University of São Paulo, BRAZIL

## Abstract

Based on the histological features and outcome, the current WHO classification separates thymomas into A, AB, B1, B2 and B3 subtypes. It is hypothesized that the type A thymomas are derived from the thymic medulla while the type B thymomas are derived from the cortex. Due to occasional histological overlap between the tumor subtypes creating difficulties in their separation, the aim of this study was to provide their proteomic characterization and identify potential immunohistochemical markers aiding in tissue diagnosis. Pair-wise comparison of neoplastic and normal thymus by liquid chromatography tandem mass spectrometry (LC-MS/MS) of formalin fixed paraffin embedded tissue revealed 61 proteins differentially expressed in thymomas compared to normal tissue. Hierarchical clustering showed distinct segregation of subtypes AB, B1 and B2 from that of A and B3. Most notably, desmoyokin, a protein that is encoded by the AHNAK gene, was associated with type A thymomas and medulla of normal thymus, by LC-MS/MS and immunohistochemistry. In this global proteomic characterization of the thymoma, several proteins unique to different thymic compartments and thymoma subtypes were identified. Among differentially expressed proteins, desmoyokin is a marker specific for thymic medulla and is potentially promising immunohistochemical marker in separation of type A and B3 thymomas.

## Background

The thymus is an integral part of the adaptive immune system, playing a major role in the production and development of lymphocytes starting at conception and continuing well into childhood. Thymoma is a rare thoracic epithelial tumor of the anterior mediastinum that occurs at a rate of 0.15 per 100,000 people (National Cancer Institute).[1] Thymoma research is also complicated by the lack of tumor cell lines and animal models. In order to identify biomarkers associated with thymoma types an archival set of formalin fixed paraffin embedded (FFPE) patient tissues was used to profile protein expression differences by the use of liquid chromatography tandem mass spectrometry (LC-MS/MS) based proteomics.

The World Health Organization (WHO) devised classification system for thymoma is summarized in Table 1. This classification system is based on cell histology and lymphocyte to epithelial cell ratio. This system divides thymomas based on cellular morphology in which those with oval or spindle shaped cells are designated as type A and those with plump or dendritic cells are designated as type B. Any thymic carcinoma is classified as type C. The thymus consists of two regions, the central medulla and the lymphocyte rich cortex on the periphery. Type A thymomas originate from the medulla and type B thymomas originate from the cortex. Studies have shown significant differences between survival and recurrence rates of the different subtypes, suggesting that the current WHO classification is useful in clinical practice.[2, 3] Subtypes AB, B1 and B2 have a large non-neoplastic lymphocyte presence that makes them easy to distinguish from subtypes A and B3 by histology. However, differentiating between type A and type B3 thymomas by histology is difficult in some cases because of the lack of T lymphocyte infiltration resulting in dependence of pathologist observation of overlapping cell shape and features. Thymomas A and B3 share similar immunohistochemical (IHC) staining patterns for panCK (cytokeratin), p63, EMA (epithelial membrane antigen) and TdT. Thymoma subtypes A and B3 represent the opposite extremes of 10 year survival at 95–100% vs. 36–40% respectively.[3, 4] B3 thymomas, have a higher metastatic potential and patients may benefit from radiation and/or chemotherapy in addition to thymectomy.[5, 6] For these reasons, a biomarker with the ability to distinguish between type A and type B3 thymomas will be clinically valuable in providing a supplementary objective diagnosis. Through the improvement of thymoma subtype diagnosis, an appropriate treatment plan is devised for the specific disease allowing for better patient outcome.

**Table 1:** Histological Features of the Thymomas.

Thymoma Subtype	% of Total Thymoma	10 Year Survival Rate	Morphological Features
A	4–7%	95–100%	-Spindle oval shaped cells without nuclear atypia -Few or none nonneoplastic lymphocytes -Encapsulated
AB	28–34%	90–100%	-Same features as type A -Rich in nonneoplastic lymphocytes
B1	9–20%	83–85%	-Resembles normal functioning thymus -Overwhelmingly rich in lymphocytes
B2	20–36%	71–83%	-Plump cells with vesicular nuclei -Heavy population of nonneoplastic lymphocytes
B3	10–14%	36–40%	-Round or polygonal epithelial cells with mild or no atypia (sheet like structure) -Very few lymphocytes

While the thymoma subtypes often share overlapping histological features (National Cancer Institute and Stanford Surgical Pathology), the clinical outcomes differ.

Previously, Sun et al. determined that proteins were differentially expressed in thymomas B1 and B2 using homogenized resection specimens.[7] Strobel et al. previously assessed the use of medullary and cortical markers to characterize thymoma subtypes.[8] Unfortunately, these markers were not observed in the A and B3 subtypes. Another study identified proteasome subunit β5t as means of distinguishing medulla vs. cortex derived A and B thymomas respectively.[9] Our study further assessed the protein expression differences of thymomas by profiling the proteome of FFPE archival tissue specimens of healthy thymus and thymomas A, AB, B1, B2 and B3. Protein expression differences were assessed through pairwise comparisons between normal thymus tissue and thymoma tumors of the five A/B subtypes. A protein expression profile was identified to differentiate lymphocyte-rich tumors from lymphocyte-poor. It was confirmed that onco-protein stathmin, identifies T lymphocyte content within the tumor rather than the thymoma itself. The proteomics analysis also revealed that desmoyokin was downregulated in subtype B3 relative to subtype A. Immunohistochemistry validation confirmed that desmoyokin was found in the medulla and not the cortex. Since type A thymomas are derived from the medulla while B type are derived from the cortex, subtypes A and B3 thymomas can be differentiated using desmoyokin IHC analysis.

This study performs the first comparative global proteomic profile of the thymus and the five different thymoma subtypes. While several of the findings are supportive of what is known of the thymus and thymomas, we propose the novel use of desmoyokin to distinguish between type A and B thymomas based on the tumors’ region of origin within the thymus.

## Methods

### Formalin Fixed Paraffin Embedded Patient Tissue Processing and Digestion

A total of 36 formalin fixed paraffin embedded patient tissues encompassing the five tumor subtypes and normal thymus tissue were profiled by shotgun proteomics. Biological replicates included 6 de-identified specimens for each diagnosis and no technical replicates were performed, ultimately generating a total of 36 data files. The sample size was selected in consideration of the statistical power as well as the time and cost associated with the preparation and mass spectrometry experiment. Data from the mass spectrometry experiment was then used to select protein candidates for orthogonal validation with immunohistochemistry using a tumor microarray consisting of 71 tumor cores. Due to low sample quality of one thymoma A sample the data set was rejected leaving 5 thymoma A data sets and 35 data sets total. Patient specimens were selected from cases ranging from the years 2001 and 2012. The age range of the patients was from 20 to 70 years with an average age of 54. Male to female ratio of the specimens used for mass spectrometry studies was 16:15 (data unavailable for 5 specimens). The patient data for the specimens in this study is detailed in S1 Table. All experimental work in this study was approved by The Ohio State University Institutional Review Board (IRB). Four serial sections from each specimen block were collected. One section was processed using a standard hematoxylin and eosin (H&E) protocol and specific tumorigenic areas were highlighted by a thoracic pathologist.[10] Tissues were dried for 14 days prior to processing and digestion.

FFPE tissues were deparaffinized using octane then rehydrated using a graded ethanol series (100%/90%/70%). Briefly, tissue slides were stained using 15% hematoxylin (Vector H-3404, Burlingame, CA, USA) then counterstained with toluidine blue (Fisher Scientific, Fair Lawn, NJ, USA). The toluidine blue was prepared as a stock solution by dissolving 500 mg of toluidine blue into 50 mL 70% ethanol. Working solution consisted of toluidine blue stock solution and 1% NaCl at a 1:9 ratio. Both hematoxylin and toluidine blue stains are compatible with mass spectrometry proteomics. Excess stain was removed with water and the tissue was dehydrated with a graded ethanol series (70%/95%/100%).[11]

Using the matching H&E stained tissue slide, the connective tissue surrounding the tumor was removed with a scalpel and residual debris washed away with ethanol. The tissue crosslinks were reversed by incubating the tissue slides in boiling water for 20 minutes followed by 2 hours at 60°C. Each recovered tissue was digested on-slide with 0.01% (w/v) Trypsin (Promega, Madison, WI, USA) dissolved in 0.5% (w/v) RapiGest (Waters Corporation, Milford, MA, USA) for 18 hours at 37°C. The digest was collected and debris removed by centrifugation. The supernatant was concentrated by vacuum centrifugation.

### LC-MS/MS Data Collection

Tryptic peptide concentration was estimated by 280nm measurement using a Nanodrop spectrophotometer and for each sample 1500 ng of peptides were loaded onto a μ-precolumn (PepMap100, C18, 5 μm, 100 Å, 0.3 x 50 mm) at a flow rate of 20 μL/min for four minutes. Peptide separation was completed using a Dionex UltiMate 3000 RSLCnano HPLC system equipped with an Easy-Spray PepMap C18 3μm 100Å 0.75x150 mm column (1.8kV, 275°C). A linear gradient was used at a flow rate of 0.3 μL/minute from 2–28% Phase B (Phase A: 0.1% formic acid and Phase B: 0.1% formic acid in acetonitrile) from 4–145 minutes followed by a column wash and equilibration period. The total instrument acquisition time was 180 minutes. Data-dependent acquisition on an Orbitrap Fusion was completed in top speed mode. Orbitrap full scans (400–1600 m/z) were completed every four seconds (AGC:400K ions; 120K mass resolution; 50ms max injection time, 1 microscan) followed by quadruple isolation (1.4 Da) for CID fragmentation. Fragment ion masses were measured in the linear ion trap (NCE: 35%, AGC: 100 ions; 250ms max injection time, parallelizable time was enabled). Ion fragmentation selection was limited to precursor ion charges 2–7 that exceeded 10K signal intensity and isolated based on dynamic exclusion parameters (25s ± 10ppm, repeat count:1).

### LC-MS/MS Data Analysis

RAW data files were analyzed directly using MaxQuant (v.1.5.2.8).[12] Peptide-spectrum matches (PSMs) were determined using the Andromeda search engine. Database searches were conducted against FASTA database composed of a UniProt human database (taxon:9606, 20198 entries, May 2015), common Repository of Adventitious Proteins (cRAP V1.0, 116 entries, http://www.thegpm.org/crap) (20326 total entries). MaxQuant search parameters included: peptide length >5 amino acids, maximum of two tryptic missed cleavage events, dynamic modification for methionine oxidation and protein N-terminal acetylation, precursor mass tolerance of 20 ppm and fragment mass tolerance of 0.5 Da. Peptide false discovery rate (FDR) was determined using a reverse decoy database. PSMs and protein level FDR were both set at 1%. The corresponding protein identifications contained a minimum of one razor peptide. Total ion current (TIC) normalization signal to noise ratio was set to 2. The majority protein identifier was used to represent any protein groups. Precursor spectral feature information was shared across 36 RAW files using the ‘match-between-runs’ feature (20/0.7 minute alignment/match). A minimum of two unmodified unique/razor peptides was required for MaxLFQ quantitation. These features are limited to the MaxQuant environment.[13]

Bioinformatic analysis was completed with the Perseus software (v.1.5.2.6) (http://www.perseus-framework.org). Due to the nature of this experimental design a series of two-tailed t-tests were used to determine differential protein expression with normal thymus being used as a common control. Reverse and contamination proteins were removed and a final protein list was produced by filtering based on a positive MaxLFQ intensity in at least three biological replicates in either cancer or normal tissue. This process was repeated five times from a common matrix of 1193 protein entries. MaxLFQ values were log_2_ transformed. Missing values were imputed under the assumption that the missing MaxLFQ intensity values are due to their corresponding peptides being present in the digest but under the limit of detection of the mass spectrometer. Missing value imputation was mimicked for low abundance proteins by bootstrapping MaxLFQ intensity values from the normal distribution of all values. As is commonly completed in this type of label-free proteomics experiment a 1.8 standard deviation downshift and 0.3 standard deviation width was used for the random reselection of data.[12–14] A two-sample Student’s t-test was used to determine statistically significant differential protein expression. To control for multiple comparisons, the p-values were adjusted using a Benjamini-Hochberg approach.[15]

### Gene Ontology Term Enrichment Analyses

Biological processes (GOTERM_BP_FAT) were investigated using the Database for Annotation, Visualization and Integrated Discovery (DAVID, v.6.7).[16–18] Gene ontology (GO) analyses were performed on significant UniProt identifiers for each pairwise comparison independently (q-value <0.20). The entire human genome (approximately 30000 genes total, DAVID default) was used to assess gene ontology term enrichment when analyzing the complete subtype datasets for high throughput global experiments. [16]

### Immunohistochemistry

Immunohistochemistry was performed on normal, thymoma A, AB, B1/B2, B3 and lung squamous cell carcinoma. Immunohistochemistry of tumor microarray (TMA) was selected as an appropriate validation since it is a routine clinical procedure. The ability to assess biomarkers through IHC facilitates potential future clinical use with existing equipment and protocols. FFPE tissues were cut at 4μm thickness on SuperFrost Plus slides. Slides were heated to 60°C for one hour, cooled, deparaffinized then rehydrated using xylene and graded ethanol solutions. Slides were quenched for 5 minutes in a 3% hydrogen peroxide aqueous solution to block endogenous peroxidase. Tissue was pretreated with Target Retrieval Solution (Dako S1699, Dako, Carpinteria, CA, USA) at pH 6.0. Tissues were stained with the Stathmin antibody (1:150, Santa Cruz sc-48362 mouse monoclonal, Santa Cruz, Dallas, TX, USA) for 60 minutes at room temperature using the Intellipath Autostainer Immunostaining System. Detection was performed using 2 step Mach 3 Mouse HRP Polymer Detection (Biocare M3M530L, Biocare, Concord, CA, USA) 20/20 minutes. The slides were counterstained with Richard Allen hematoxylin (Thermo Scientific, Kalamazoo, MI, USA), dehydrated with graded ethanol solutions, cleared with xylene and coverslipped. A tumor microarray consisting of 71 total biopsy tissue cores (9 thymoma A, 18 thymoma AB, 9 thymoma B1/B2, 15 thymoma B3, 3 thymic carcinoma, 7 normal thymus, 5 lung squamous cell carcinoma and 5 lymph node) was used for IHC staining. The TMA specimens were derived from patients ages 20–69 with an average age of 54. Male to female ratio of the specimen sources was 29:24 (data unavailable for some specimens). The clinical data associated with samples used for the thymoma tumor microarray is summarized in S2 Table. The tumor microarray was scanned digitally then viewed for analysis using the Aperio Microscopy Viewer. Similarly, desmoyokin IHC scans were performed using the AHNAK/desmoyokin antibody (1:400, Atlas Antibodies HPA026643 rabbit polyclonal, Atlas Antibodies, Stockholm, Sweden). Antibodies to terminal deoxynucleotidyl transferase (TdT) diluted at 1:25 were used to stain a tumor microarray to serve as a control to define the presence of immature T lymphocytes in patient tissues.

## Results

### Protein in Thymus vs. Thymoma Comparison

**Fig 1:**
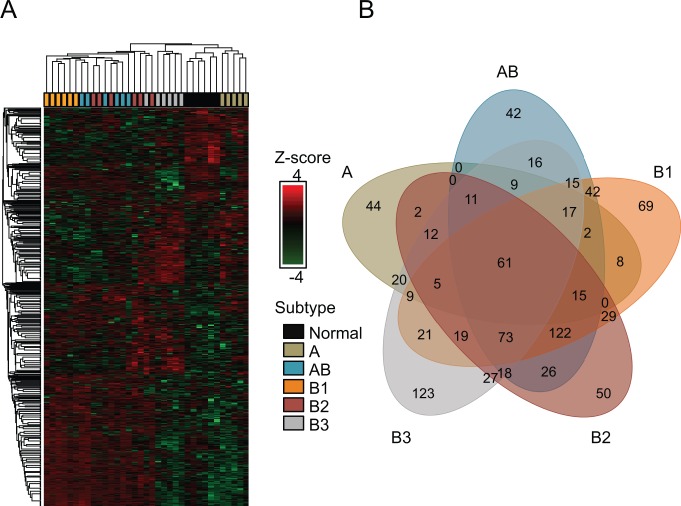
Clustering and Venn Diagram of Significant Proteins (q-value <0.20). **A.** Unsupervised clustering of the 35 individual thymus samples reveal distinctive clustering within different subsets of normal and tumorous thymus tissue. This indicates the interpatient variability does not exceed intersubtype variability. **B.** Proteins identified in thymoma samples were matched against normal thymus tissue to obtain a fold change in protein expression. Proteins displaying a significant fold change of q-value 0.20 were compared between the different tumor subtypes.

A total of 1193 proteins were identified in our proteomic analysis of normal thymus and tumor subtypes. Multiple pairwise comparisons were completed to determine differential protein expression between normal thymus tissue and each thymoma subtype. After stringent filtering: 918, 999, 957, 1034 and 982 matched protein identifications (S3 Table) were retained for A, AB, B1, B2 and B3 vs. normal thymus, respectively. There were 1,168 unique proteins in the union of these datasets. Implementing a significance cut-off: 215, 469, 507, 470 and 456 proteins were determined to be differentially expressed in subtypes A, AB, B1, B2 and B3, respectively. The union of these datasets yielded a total of 907 unique proteins. The union of significantly differentially expressed proteins were evaluated by hierarchical clustering and principle component analysis (Fig 1A and S1 Fig). There were 61 proteins that were differentially expressed in all five tumor subtypes when compared to normal thymus tissue (Fig 1B and S4 Table). Of these 61 proteins, 7 proteins belonging to the collagen family are downregulated in thymomas in relation to normal thymus (Table 2). The collagens CO1A1, CO1A2, CO3A1, CO5A1, CO6A1, CO6A2 and CO6A3 were distinguished by 17, 18, 7, 2, 20, 24 and 97 unique peptides respectively.

**Table 2:** Collagen Proteins Differentially Expressed in Thymomas from Normal Thymus.

		NvA		NvAB		NvB1		NvB2		NvB3	
Protein Name	UniProt Header	q-value	Log_2_ Fold Change	q-value	Log_2_ Fold Change	q-value	Log_2_ Fold Change	q-value	Log_2_ Fold Change	q-value	Log_2_ Fold Change
CO1A1 Collagen alpha-1(I)	P02452	0.086	-1.705	0.024	-2.119	0.017	-1.816	0.004	-3.333	0.124	-1.659
CO1A2 Collagen alpha-2(I)	P08123	0.017	-2.807	0.023	-2.275	0.019	-1.985	0.005	-3.261	0.023	-2.733
CO3A1 Collagen alpha-1(III)	P02461	0.104	-2.235	0.005	-4.887	0.006	-2.915	0.009	-4.414	0.118	-2.510
CO5A1 Collagen alpha-1(V)	P20908	0.040	-6.066	0.181	-3.392	0.022	-5.298	0.014	-5.711	0.124	-4.074
CO6A1 Collagen alpha-1(VI)	P12109	0.080	-0.842	0.016	-1.075	0.011	-1.614	0.086	-1.374	0.040	-1.692
CO6A2 Collagen alpha-2(VI)	P12110	0.072	-0.719	0.014	-1.087	0.018	-1.607	0.091	-1.556	0.038	-1.718
CO6A3 Collagen alpha-3(VI)	P12111	0.066	-0.728	0.023	-0.907	0.017	-1.350	0.088	-1.341	0.016	-1.558

Seven collagen proteins were significantly differentially expressed between normal thymus and thymoma. The associated fold changes and significance are listed in this table.

### Proteins in Thymoma Subtypes Comparison

Cluster analysis of the 35 samples showed two distinct clusters. Cluster one consists of subtypes AB, B1 and B2, while cluster two consists of subtype A and B3 and normal thymus. Furthermore, cluster two can be sub-clustered into subtypes A and B3 and normal thymus. Cluster one was unable to be sub-clustered due to the heterogeneous proteomic signature from intermixed T lymphocytes. The presence of T lymphocytes was confirmed in both H&E and IHC imaging. DAVID was used to compare the gene ontology terms enriched in each thymoma subtype (S5 Table). A qualitative analysis of the proteins identified in the different thymoma samples was visualized as a Venn diagram (Fig 1B) and the proteins in each intersection are provided in S4 Table. It is notable that 122 proteins were differentially expressed in thymomas AB, B1 and B2 (intersection), the largest overlap in this analysis.

### Contribution of Lymphocyte Component to the Tumor Protein Profile

**Fig 2:**
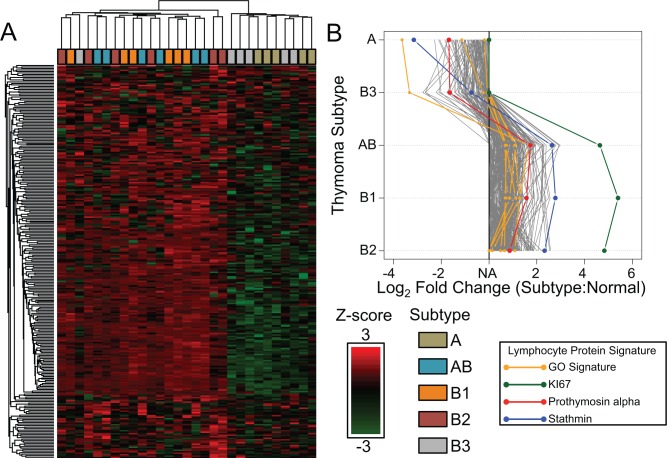
Proteomic Profile Contribution of T Lymphocytes. **A.** Heatmap plot of the 263 T lymphocyte candidates across the 29 thymoma samples produced after MaxLFQ values were z-normalized. Hierarchical clustering was performed using Euclidian distance and average linkage using the Perseus software. **B.** Line plot of log2 fold change by subtype of the pattern associated with T lymphocyte infiltration. Prothymosin alpha (red) is a known T lymphocyte associated protein found in the thymus. Proteins matching DAVID GO Term T Cell Differentiation (GO:0030217) were highlighted in orange. Stathmin (blue) a protein assessed for subtype diagnosis potential. Ki67 (green) is a known cell proliferation marker.

**Fig 3:**
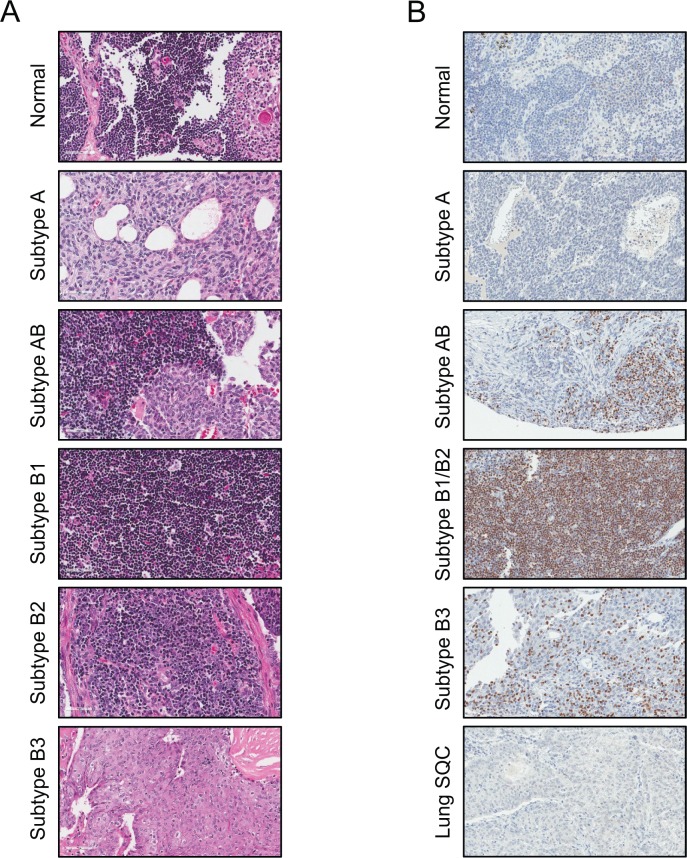
Intratumoral Lymphocytes Throughout Thymoma Subtypes. **A.** Histology of normal thymus and the thymoma types: A, AB, B1, B2 and B3. Tissue samples were stained with hematoxylin and eosin. Images were captured at 400x magnification. **B.** The different thymus specimens were stained with TdT (terminal deoxynucleotidyl transferase) an established maker for developing T lymphocytes (40x magnification).

The WHO categorization of thymoma subtypes assesses T lymphocyte content. Specifically, subtypes AB, B1 and B2 are lymphocyte-rich in contrast to lymphocyte-poor A and B3. The data from this study demonstrated a pattern, which allowed for identification of candidate T lymphocytes proteins found within thymoma samples. Prothymosin alpha, precursor of thymosin α1, is produced in the thymus and cleaved to regulate T lymphocyte proliferation.[19, 20] Thus, prothymosin alpha was selected as a T lymphocyte associated marker to establish a pattern of expression for potential lymphocyte markers. The log_2_ fold changes of prothymosin alpha in AB, B1 and B2 in comparison to normal thymus were 1.73 (q-value 0.0107), 1.57 (q-value 0.0120) and 0.862 (q-value 0.0902) respectively. Downregulation of prothymosin alpha was observed in types A and B3 thymomas, with significant changes observed in B3, -1.68 (q-value 0.247) and -1.65 (q-value 0.0262) respectively. Proteins following this pattern of exhibiting downregulation or no change in subtype A and B3 thymomas while exhibiting upregulation in A, B1 and B2 thymomas were assessed as T lymphocyte proteins candidates (S6 Table). A total of 263 proteins followed this expression pattern and was subsequently analyzed using cluster analysis. A heatmap of the 263 proteins across the 29 thymoma samples was produced after the MaxLFQ values were z-normalized (Fig 2A). Cluster analysis revealed distinctive clustering based on T lymphocyte infiltration status with the exception of one thymoma B3 sample. Proteins with sample variation were observed and visualized in the top and bottom of the heatmap. GO term molecular functions were assessed by DAVID (S7 Table) using the total detected protein set as background. From the protein candidates, six of the proteins from this list showed function associated with T lymphocytes in the GO term T Cell Differentiation (GO:0030217) (p-value 4.89 x 10^−2^): CD3D, CD3E, ZAP70, BCL11B, LCK, and RPL22. The T lymphocyte candidate proteins were visualized in a line plot of log_2_ fold change, highlighting proposed T lymphocyte protein expression pattern between subtypes using prothymosin alpha and the proteins associated with T cell differentiation (Fig 2B). Lymphocyte infiltration status of the different thymoma types was validated using anti-terminal deoxynucleotidyl transferase (TdT) immunohistochemistry (Fig 3).

**Fig 4:**
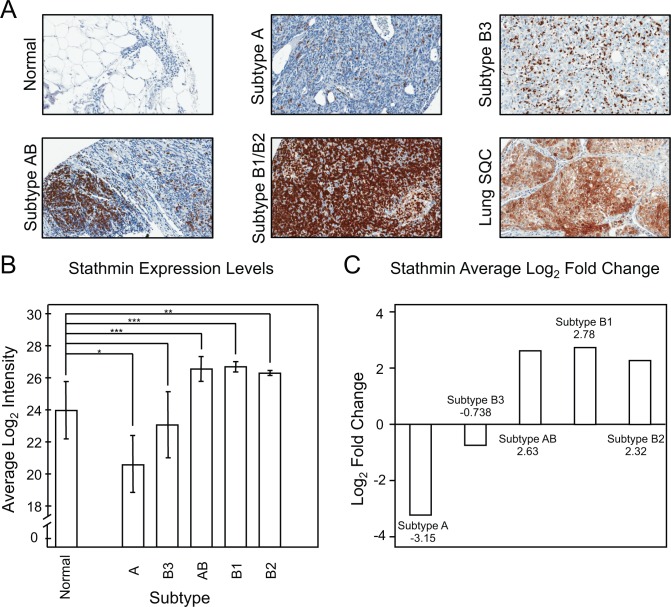
**Changes in Stathmin Expression in Different Thymoma Subtypes: A.** Heavy stathmin staining was noted in the T lymphocytes of AB, B1 and B2 as well as in the epithelial derived lung squamous cell carcinoma tumor (40x magnification). **B.** Bar graph displaying the intensity quantitation of stathmin for the thymus and thymoma samples. Standard deviation is denoted in the error bars. (q- values: *** <0.05, ** 0.05–0.10 and * 0.10–0.20) **C.** Log_2_ fold changes of stathmin expression of the different thymomas in comparison to normal thymus.

The established onco-protein stathmin was evaluated as a potential marker for distinguishing thymoma subtypes.[21–23] Stathmin was found to be upregulated in thymoma AB, B1 and B2 in relation to normal thymus tissue (Fig 4B and 4C). Immunohistochemical staining of thymoma tissues with anti-stathmin showed that the protein was localized to the lymphocytes infiltrating the thymus tissue (Fig 4A). In contrast, lung squamous cell carcinoma, an epithelial tumor used as a positive staining control, stained heavily for stathmin in the epithelial cells. Thus, data indicates that the elevation of stathmin detected in AB, B1 and B2 are T lymphocyte in origin and not from the epithelial tumor.

### Proteins associated with type A and B3 thymomas

Pairwise log_2_ fold comparison of A and B3 thymomas was performed to assess for candidates that differentiate between the two subtypes, and 90 proteins were determined to show significant differences in expression (S3 Table). Gene ontology analysis through DAVID revealed proteins associated with the terms anti-apoptosis (GO:0006916, p-value 1.50 x 10^−3^), negative regulation of apoptosis (GO:0043066, p-value 1.90 x 10^−2^), negative regulation of programmed cell death (GO:0043069, p-value 2.10 x 10^−2^) and negative regulation of cell death (GO:0060548, p-value 2.10 x 10^−2^) (S8 Table). These GO terms consists of an overlapping set of 7 proteins: annexin 1, annexin 4, alpha B crystallin, DDAH (dimethylarginine dimethylamaminohydrolase 2), heat shock protein 70, heat shock protein 90 and NFKB1 (nuclear factor of kappa light polypeptide gene enhancer in B-cells 1) (Table 3).

**Table 3:** Anti-apoptotic Proteins were Differentially Expressed in Thymomas A and B3.

Protein Name	UniProt Header	q-value	Log2 Fold Change (A/B3)
Annexin 1	P04083	0.138	-1.784
Annexin 4	P09525	0.121	-0.998
Alpha b crystallin	P02511	0.087	-4.181
DDAH 2	O95865	0.084	-2.019
HSP 70	P38646	0.023	0.798
HSP 90B1	P14625	0.135	-0.726
NFKB1	P19838	0.072	1.701

Gene ontology analysis determined that anti-apoptotic proteins in the set of 90 proteins showing significant differences in protein expressions thymomas A and B3. Fold changes and significance are listed in this table.

### Desmoyokin is associated with medulla of normal thymus and type A thymomas

**Fig 5:**
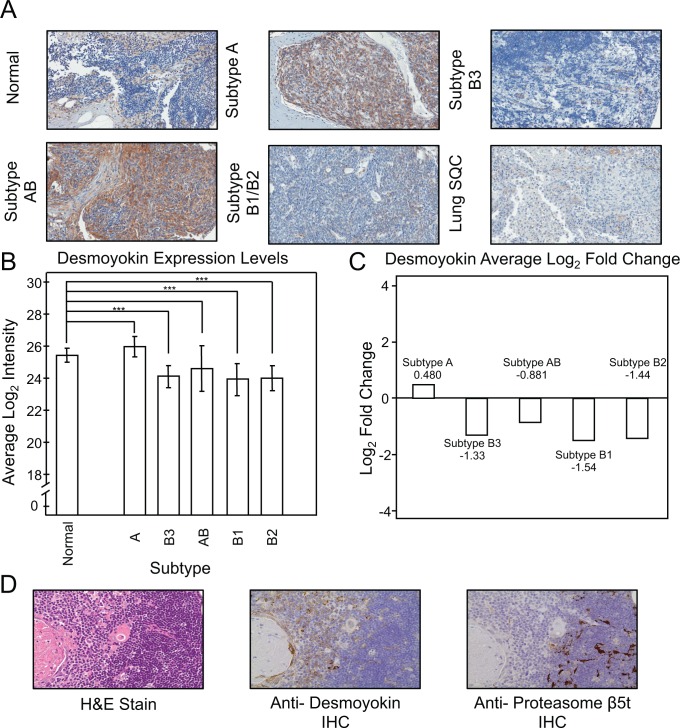
Desmoyokin is Differentially Expressed in and A and B Thymomas. **A.**Thymus tissue of A, AB, B1, B2 and B3 were stained with anti- desmoyokin antibody. The type A thymomas (A and AB) stained heavily while the type B (B1, B2 and B3) and normal thymus tissue expressed low amounts of the protein (40x magnification). **B.** Bar graph displaying the intensity quantitation of desmoyokin for the thymus and thymoma samples. Standard deviation is denoted in the error bars. (q- values: *** <0.05, ** 0.05–0.10 and * 0.10–0.20) **C.** Log_2_ fold changes of desmoyokin expression of the different thymomas in comparison to normal thymus. **D.** Normal thymus tissue is stained with hematoxylin and eosin, anti- desmoyokin and anti- proteasome β5t.

Desmoyokin, a protein encoded by the AHNAK (neuroblast differentiation-associated) gene, was a protein of interest exhibiting a log_2_ fold change of -1.81 (q-value 0.0668) in B3 thymomas in relation to A thymomas with known associations with cancer. Expanding the analysis across all tumor subtypes, desmoyokin was determined to be significantly downregulated in B1, B2 and B3 in comparison to normal thymus, while it was not determined to be significantly different in A and AB tissues from normal thymus tissue (Fig 5B and 5C). Immunohistochemistry staining of thymoma and normal thymus tissue using an antibody to desmoyokin confirmed that its expression was increased in subtype A (Fig 5A). A tumor microarray consisting of 36 viable cores was stained for desmoyokin expression and evaluated as negative, low or high. These 36 cores were subtyped and 8 were subtype A, 7 were B1, 10 were B2 and 11 were B3. AB thymoma cores were excluded due to the heterogeneity of the tumors. Results of this evaluation were in agreement with mass spectrometry data as the tumor microarrays for type A thymoma stained significantly higher for desmoyokin than Type B thymomas. The chi-square tests for associations between thymoma subtypes and desmoyokin resulted in a Pearson chi squared value of 15.9, indicating that its abundance was significantly associated with subtype (p-value 0.014). Association between thymoma A vs. grouped thymoma B (B1, B2 and B3) and desmoyokin was also performed. The resulting Pearson chi squared value obtained was 15.8, demonstrating significance (p-value 0.00038). We compared H&E, anti-desmoyokin and anti-proteasome subunit β5t stained normal thymus tissue (Fig 5D). From this staining we observed desmoyokin staining exclusively within the thymus medulla and found proteasome subunit β5t staining uniquely within the cortex, as expected.[9] In summary, we show that the protein desmoyokin is a potentially useful protein marker in difficult cases for differentiating the diagnosis in thymomas A and B3 clinically.

## Discussion

This study is the first global proteomic profiling of normal thymus and thymomas through the use of archival patient tissues and LC-MS/MS. In this study we were able to identify protein profiles of the different disease subtypes. We identified desmoyokin as a protein associated with the thymus medulla, hence, a novel identification marker for type A thymomas.

It was determined that 61 proteins showed significant changes in expression level from normal thymus tissue. Seven of these 61 proteins belong to the collagen family, all of which were at lower abundance in thymomas. Changes in collagen in cancer have been extensively studied and reviewed [24]. During the cancer development process, remodeled collagen I, III, IV is deposited while collagen that forms a physical barrier is degraded promoting tumor cell proliferation and invasion. Dysregulation of collagen levels in the stroma can be oncogenic. Existing studies have shown that COL1A1 (encoded by the collagen type 1 alpha 1 gene) has some predicative value in tumor diagnosis and prognosis where it has been found to be upregulated and downregulated in oral squamous cell carcinoma and hepatocellular carcinoma respectively.[25–27] Another explanation for the observed collagen changes is the reversion of the atrophied adult thymus composed mostly of adipocytes and stroma. However, few studies have been conducted on collagen composition changes in association with the thymus. To date, this is the first evidence of collagen changes in association with thymomas. Future study on the changes in collagen distribution factors in thymoma may further elucidate its role in tumorigenesis.

Hierarchical cluster analysis revealed a distinctive separation of the different thymoma subtypes into two groups, with AB, B1 and B2 grouping together while A, B3 and normal thymus formed a second group. The proteomic results in this study were then validated using IHC. We concluded that lymphocyte presence contributed significantly to the clustering results. The histology based subtype classification devised by the WHO defines AB, B1 and B2 thymomas as possessing heavy non-neoplastic T lymphocyte infiltration. Thymoma AB contains large pockets of lymphocytes within the epithelial cell structure in the type B-like regions. To establish a pattern associated with T lymphocyte content, a marker with a known relationship to T lymphocytes with specificity to the thymus was needed. Prothymosin alpha, the precursor to thymomsin α1, was selected for this purpose. Thymosin α1 is a peptide signaling molecule mainly produced in the thymus that plays a role in the proliferation and maturation of T cells.[28, 29] Due to the immersion of the biopsy tissue during the FFPE preservation process, free active biomolecules were likely lost. However, thymosin α1 can be monitored through its uncleaved precursor prothymosin alpha found inside epithelial tumor cells. The use of prothymosin alpha as a surrogate for thymosin α1 was previously demonstrated in literature for radioimmunoassays.[20, 30] Our proteomics analysis of FFPE archival tissue revealed that in comparison to normal thymus tissue thymoma of AB, B1 and B2 types show an increase in prothymosin alpha while thymomas A and B3 show a lower level of prothymosin alpha, consistent with expected levels of T lymphocytes. Thus, using the expression pattern of prothymosin alpha as a template, candidates for other T lymphocyte associated proteins were identified. This finding also offers a potential explanation that tumor tissue may be abnormally producing signaling factors leading to the proliferation of the infiltrating T lymphocytes.

A total of 263 unique proteins were identified as T lymphocyte associated protein candidates. The clustering of this data set revealed segregation between thymoma samples associated with the degree of lymphocyte infiltration with the exception of one B3 outlier. As thymoma B3 originates from the thymus cortex, the site of lymphocyte maturation, a varying amount of T lymphocytes may be present. It can be hypothesized that the outlier sample possessed a higher lymphocyte content resulting in the observed grouping. Through DAVID GO term analysis 6 of the proteins expressed multiple hits in functions associated with T lymphocytes in the GO term T cell differentiation. CD3 delta (CD3D) and epsilon (CD3E) are chains of the CD3 complex. CD3 is a component of the T-cell receptor complex, which also consists of the ζ chain and either αβ T-cell receptor or γδ T-cell receptor. The T-cell receptor complex conveys signals from the extracellular T cell receptor to intracellular signaling pathways.[31, 32] CD3D is required for transition of the T cell from the double positive to the single positive stage.[33] CD3E is necessary for progression of T cells past the double negative stages. The loss of CD3E leads to absence of mature double and single positive thymocytes implicating its role in pre-TCR development.[34–36] Brodeur et al. showed that the intracytoplasmic tail of CD3E is essential for double negative to double positive transition.[37] Tyrosine kinase LCK (LCK), tyrosine kinase ZAP-70 (ZAP70) and B-cell lymphoma/leukemia 11B (BCL11B), play roles in the differentiation and survival of T lymphocytes.[38–43] LCK plays a role in pre-TCR signaling at the double negative 3 (DN3) transition to double negative 4 (DN4) for the commitment of alpha-beta T cells development.[44–46] Ribosomal protein, RPL22 is also essential for the alpha-beta differentiation of T cells during the DN3 stage.[47–49] It is also notable that Ki67, a well-established proliferation marker, was found in all 18 of the AB, B1 and B2 samples but not in any of the A and B3 samples. In further support of the importance of lymphocyte contribution to the protein profile, a high degree of overlapping proteins was found among thymoma AB, B1 and B2 samples which are characteristically lymphocyte-rich, as confirmed by TdT IHC staining.

Stathmin, a protein involved in the regulation of microtubule dynamics, has been well established as a marker for high cell proliferation rate.[50, 51] Stathmin is over-expressed in several different cancer types.[23, 52–55] High levels of stathmin have been associated with poor prognosis in multiple cancer types and resistance to drugs stabilizing microtubules, such as taxane.[56] Furthermore, depletion of stathmin leads to cell cycle arrest in the G2 phase and apoptosis.[57] Due to the known correlation between stathmin expression level and other epithelial cancers, the differential expression of stathmin detected between the thymoma tumor types was further investigated using IHC. The IHC staining showed that the stathmin was localized to the T lymphocytes infiltrating the biopsy tissue, leading to the conclusion that stathmin is a lymphocyte associated protein. The positive staining of stathmin in T lymphocytes is supported by previous studies, as stathmin plays a critical role in the activation of T lymphocytes as a regulator of cell polarization and T lymphocyte migration from the vascular compartment across tissue barriers.[57, 58] The use of stathmin to distinguish the different thymoma subtypes is limited due to its staining of lymphocytes as opposed to the tumorous epithelial cells themselves, as CD4 and CD8 are well-established IHC targets for T lymphocytes.[59]

Comparison of the proteomic profiles between A and B3 revealed 90 proteins to be significantly expressed differentially between the subtypes. Of these 90 proteins most were involved in biological functions not obviously associated with cancer including sugar and amino acid metabolism. Seven of these proteins are involved in anti-apoptotic processes. It is known that some proteins may serve roles as either onco-proteins or tumor suppressors in different diseases. Annexins 1 and 4 were found to be significantly downregulated in thymoma B3 relative to thymoma A. Interestingly, decreases in Annexin 1 expression have been correlated to esophageal and prostate cancer severity while showing the opposite relationship with pancreatic cancer.[60, 61] Annexin 4 while typically found over expressed in most tumors was found to have the opposite relationship in prostate cancer.[62, 63] HSP70 expression is also higher in thymoma B3 than A, consistent with its activity promoting cell survival.[64, 65] We found that NFKB1, which controls genes associated with processes such as apoptosis inhibition and cell cycle progression, was expressed significantly higher in B3 thymoma.[66–68] DDAH2 is a protein that was found relatively expressed at a greater level in type A thymoma than in type B3. There exists evidence that DDAH2 expression may be up or down regulated in different tumor types, as exemplified by ovarian carcinoma and oral squamous cell carcinoma respectively.[69, 70] High levels of proteins HSP90b1 and αB-crystallin are typically associated with cancer progression.[62, 71–73] Surprisingly, these proteins were found to be significantly more highly expressed in thymoma A over thymoma B3, despite B3 being the more severe disease. Differences in the site of origin of thymoma A and B3 may play a role in these unexpected results. Since there is no previous work on these proteins in the context of thymoma, further study and validation of these proteins is required to understand their specific roles in the disease process and to assess their potential as diagnostic and/or prognostic markers.

The search for a protein maker to differentiate types A and B3 thymomas for potential use in clinical diagnosis led to the investigation of desmoyokin, the protein encoded by the AHNAK (neuroblast differentiation- associated) gene. Protein candidates were selected for further evaluation based on highest absolute fold change difference between thymomas A and B3. Candidates included αB-crystallin (log_2_−4.18 fold change), protein s100-a10 (log_2_−2.13 fold change), protein s100-a6 (log_2_−2.05 fold change), desmoyokin (log_2_−1.81 fold change) and galectin 7 (log_2_ 3.58 fold change). The protein candidates were then further evaluated through assessment of fold change between all thymoma subtypes and normal thymus tissue for expression level patterns. Protein s100-a10 and galectin 7 were only present in A and B3 thymomas respectively when matched with normal thymus tissue. Protein s100-a6 was found to be elevated in A, AB, B2 and B3. While s100-a6 is a known tumor marker, its enhanced expression in four of the thymoma subtypes excluded it as a useful marker to distinguish between the different subtypes.[74] αB-crystallin showed elevated expression level in type A thymoma while decreased expression in AB, B1, B2 and B3, significantly in B1 and B3.

Desmoyokin was significantly downregulated in all three B thymomas while exhibiting little changes in type A and AB, supporting the fact that the protein is associated with the epithelial cell derived thymoma tumors as opposed to T lymphocytes. Furthermore, we hypothesized that desmoyokin serves as a marker distinguishing the thymus medulla from the cortex. Desmoyokin was selected for further study through IHC of tumor microarray due to its significance and expression level pattern between the A and B thymoma subtypes.

Comparison of desmoyokin staining of thymus tissue with staining by hematoxylin and eosin, and anti-proteasome subunit β5t revealed that regions were stained either exclusively with desmoyokin or with proteasome subunit β5t. Proteasome subunit β5t is a protein previously described as expressed exclusively in the thymic cortical epithelial cells.[9] Use of proteasome subunit β5 was selected as a negative control since has been shown to be unique to the cortex. It is notable that Keratin 5 and Keratin 8 expression are also used as markers for medulla and cortex but other studies have cast doubt on their specificity.[75–78] Our proteomic data showed no significant difference in Keratin 5 or Keratin 8 expression in A vs. B3 thymoma subtypes. Other markers that have been associated with cortical thymus epithelial cells, such as LY51 and CD205, were also not observed in our proteomic results. Thus, it was concluded that desmoyokin is a marker present in the thymus medulla but not the cortex, providing objective identification of thymus cortex and medulla. Since type A thymomas are derived from medullary thymus tissue and type B thymomas are derived from cortical tissue, desmoyokin serves as a marker distinguishing tumor subtypes based on tissue of origin. Whether desmoyokin observed in the thymus plays a role in tumorigenesis or the reason for this alteration has yet to be determined. The desmoyokin protein may be downregulated in cancers.[79, 80] Previous studies have shown that its levels negatively correlate with cell division rate. This protein is post-translationally modified through phosphorylation of serine and threonine, with phosphorylation occurring in actively growing cells. A study by Lee et al. showed that desmoyokin can function as a tumor suppressor through regulation of the TGFβ/Smad pathway leading to cell cycle arrest.[81] This study showed that desmoyokin expression was significantly downregulated in B subtype thymomas compared to the A subtype thymomas and normal thymus tissue. The A subtype thymomas did not show a significant difference in desmoyokin expression when compared to normal thymus tissue. It is clear that much remains to be learned about the desmoyokin protein in the context of cancer.[81] One potential future course of study is to study the phosphorylation states of the desmoyokin protein in thymomas to assess for possible correlation with abnormal cell growth patterns. Thus, this protein is a target for future study not only as a marker for clinical function to distinguish between thymoma types A and B3 but also to further elucidate the changes associated with tumorigenesis of thymomas from the different regions of the thymus.

## Conclusion

This study is the first global proteomic characterization of all epithelial derived thymoma subtypes compared to normal thymus, overcoming the challenges associated with studying a disease with no representative cell line or animal model. Challenges due to the rarity of this disease were addressed through the utilization of FFPE tissues collected over two decades. The resulting proteomic characterization has led to the discovery of interesting potential biomarker proteins: desmoyokin and stathmin. This study revealed that desmoyokin was downregulated in thymus cortex tissue, the site of origin for subtype B thymomas but not subtype A thymomas. This protein may allow for differentiation between thymomas based on their site of origin. The stathmin protein ultimately proved to be unsuitable for a disease biomarker to separate thymoma subtypes, but its changes in abundance across these thymomas supports existing knowledge of associated lymphocytic infiltration. These findings on desmoyokin and stathmin have opened the possibility for several future studies exploring the role of the immune system, the cell cycle, and cell motility pathways in the development of this disease. The molecular data from this study also provide a new opportunity for comparison to other cancers in an effort to elucidate the process of tumorigenesis.

## Supporting Information

S1 Fig Principal Component Analysis of Thymoma Subtypes. **A.** Scatterplot of the first two principal components of the PCA **B.** Scree plot of the first eight principal components.
(EPS)

S1 Table Patient Details for LC-MS/MS Experiment. Patient age and gender for tissue specimens used for LC-MS/MS proteomics experiment.
(XLSX)

S2 Table Patient Details for Tumor Microarray. Patient age and gender for tissue specimens used for tumor microarray studies.
(XLSX)

S3 Table Imputed Protein Lists. List of detected thymoma proteins and their associated detected intensities and statistics in relationship to normal thymus. Note an imputed value corresponding to low end detection values were used for proteins not detected in individual samples.
(XLSX)

S4 Table Intersections of Proteins Identified Between Subtypes Identifications of protein components of each intersection in the Venn diagram of Fig 1B. (Attached Excel Spreadsheet S5 Table)
(XLSX)

S5 Table DAVID Gene Ontology Analysis. DAVID was used to analysis proteins lists significantly (q-value <0.20) different between tumor and normal thymus tissue to assess Gene Ontology for protein similar in function.
(XLSX)

S6 Table T Lymphocyte Associated Protein Candidates. List of thymoma proteins determined to be potential T lymphocyte associated proteins and their associated statistics in comparison to normal thymus.
(XLSX)

S7 Table DAVID Gene Ontology Analysis of T Lymphocyte Associated Protein Candidates. (XLSX)

S8 Table DAVID Gene Ontology Analysis of Protein Showing Significant Differential Expression AvB3. (XLSX)
